# Current and Emerging Pharmacological Therapies for Hypertriglyceridemia

**DOI:** 10.3390/ijms27083573

**Published:** 2026-04-16

**Authors:** Ibrahim S. Alhomoud

**Affiliations:** Department of Pharmacy Practice, College of Pharmacy, Qassim University, Buraydah 51452, Qassim, Saudi Arabia; i.alhomoud@qu.edu.sa

**Keywords:** hypertriglyceridemia, cardiovascular risk, apolipoprotein C-III inhibitors, ANGPTL3 inhibitors, olezarsen, plozasiran, evinacumab, pegozafermin

## Abstract

Hypertriglyceridemia is a well-recognized contributor to residual atherosclerotic cardiovascular disease risk and a predisposing factor for acute pancreatitis. Despite the availability of pharmacologic agents and lifestyle interventions, patients with severe and refractory hypertriglyceridemia often fail to achieve adequate control. Recent advances in the molecular understanding of triglyceride metabolism have driven the development of targeted therapies that selectively modulate key regulatory pathways. This study sought to provide an overview of triglyceride regulation, the atherogenic role of remnant lipoproteins, and clinical evidence of emerging triglyceride-lowering therapies. Lipoprotein metabolism is regulated by a complex network of regulatory proteins that include lipoprotein lipase (LPL), apolipoproteins such as apolipoprotein C-III (ApoC-III), and angiopoietin-like proteins (ANGPTLs). Targeting these proteins in the metabolic cascade has shown promising results in reducing triglyceride levels. Emerging therapies such as antisense oligonucleotides (ASOs) and small interfering RNA (siRNA) directed against ApoC-III (volanesorsen, olezarsen, and plozasiran), inhibitors of ANGPTL3 (evinacumab and zodasiran), and fibroblast growth factor 21 (FGF-21) analogs (pegozafermin) have demonstrated substantial triglyceride-lowering efficacy. These agents have achieved reductions in triglyceride levels of up to 80% in clinical trials. Additionally, preliminary evidence suggests that these agents may also reduce the incidence of acute pancreatitis and improve cardiometabolic risk profiles, although dedicated trials are still needed to confirm these outcomes. The therapeutic landscape for hypertriglyceridemia is rapidly evolving. Integrating these novel agents into clinical practice will require individualized treatment plans, sustained lifestyle modification, and careful safety monitoring.

## 1. Introduction

Triglyceride elevations are classified as moderate (150–499 mg/dL), severe (500–999 mg/dL), or extremely severe (≥1000 mg/dL). According to data from the National Health and Nutrition Examination Survey (NHANES) analysis from 1999 to 2018, approximately 26% of United States adults have moderate hypertriglyceridemia, while the prevalence of severe and extremely severe elevations is 0.9% and 0.2% of individuals, respectively [1]. However, there is no universally accepted definition of hypertriglyceridemia, as classification criteria vary slightly among major medical societies (Table 1) [2,3,4,5]. This variability in triglyceride thresholds presents challenges in both clinical research and practice, as it may lead to inconsistent patient classification, affecting eligibility criteria and the interpretation of clinical trial outcomes.

The etiology of hypertriglyceridemia is heterogeneous, ranging from inherited disorders to diverse secondary causes that include dietary factors, pharmacologic agents, excessive alcohol intake, and comorbid clinical conditions [2]. Insulin resistance and uncontrolled diabetes, for instance, lead to increased hepatic secretion of very-low-density lipoproteins (VLDL), ultimately reducing lipoprotein lipase (LPL) activity and compromising triglyceride clearance [4]. These pathophysiological pathways contribute to the accumulation of atherogenic particles in the circulation [6]. Even with optimal lowering of low-density lipoproteins (LDL) cholesterol, patients may experience residual cardiovascular risk due to the accumulation of triglyceride-rich remnant lipoproteins [7,8]. Therefore, it is essential to systematically evaluate hypertriglyceridemia, address potential secondary causes, and implement targeted therapeutic strategies according to the severity of the condition [3].

Lifestyle modification remains the cornerstone of therapy, with specific recommendations for limiting added sugars, alcohol intake, and total dietary fat according to triglyceride levels (Table 2). Importantly, regardless of baseline triglyceride levels, all patients are advised to engage in regular physical activity and weight loss [3]. This is particularly important for those with increased adiposity, as achieving a 5–10% reduction in body weight can lower triglyceride levels by approximately 10–30%, and in some cases, reductions of up to 70% have been reported [3,9,10]. Furthermore, pharmacologic agents with triglyceride-lowering effects can be considered based on clinical indications and the patient’s overall cardiovascular risk profile [3]. Emerging therapies that target novel pathways involved in triglyceride metabolism offer additional options for patients with severe or refractory hypertriglyceridemia.

This narrative review is based on a literature search conducted in PubMed, Scopus, and ClinicalTrials.gov to identify relevant studies on triglyceride metabolism and pharmacological therapies. The search included articles published up to 2025 using keywords such as “hypertriglyceridemia”, “ApoC-III inhibitors”, “ANGPTL3 inhibitors”, “siRNA”, “antisense oligonucleotides”, and “FGF21 analogs”. Studies were selected based on their relevance to the scope of this review, with priority given to clinical trials, meta-analyses, and recent high-impact publications. Non-English articles and studies not directly related to triglyceride metabolism or its pharmacological management were excluded.

The purpose of this review is to summarize the current understanding of triglyceride metabolism and the pathophysiological impact of remnant lipoproteins, with particular emphasis on recent advances in pharmacologic therapies evaluated for the management of hypertriglyceridemia.

## 2. Metabolic Biochemistry of Triglycerides

Triglycerides consist of a glycerol backbone esterified with three fatty acids. Triglycerides in the human body are derived from two main sources: exogenous triglycerides synthesized in enterocytes following dietary lipid absorption, and endogenous triglycerides synthesized primarily in the liver from carbohydrates and fatty acids [13]. Within enterocytes, newly synthesized triglycerides are assembled with apolipoprotein B-48 (ApoB48) to form pre-chylomicrons in the endoplasmic reticulum, a process dependent on microsomal triglyceride transfer protein (MTTP). These pre-chylomicrons are subsequently processed in the Golgi and secreted into the lymphatic system, where they mature into chylomicrons [14,15]. Similarly, in the liver, endogenously synthesized triglycerides are packaged with apolipoprotein B-100 (ApoB100) to form VLDL. Both chylomicrons and VLDL serve as major vehicles for triglyceride transport in the circulation [16]. Disruption of these pathways can lead to significant metabolic derangements and a spectrum of clinical consequences affecting nutrient absorption, hepatic function, and other organ systems [17].

LPL is essential for triglyceride metabolism in chylomicrons and VLDL, in which it hydrolyzes their triglyceride content and generates cholesterol-rich remnant lipoproteins [18]. The activity of LPL is tightly regulated by several apolipoproteins. Apolipoprotein C-II (ApoC-II) serves as an obligatory cofactor that activates LPL, whereas apolipoprotein C-III (ApoC-III) inhibits LPL-mediated hydrolysis. Additionally, apolipoprotein A5 (ApoA5) facilitates the interaction between LPL and triglyceride-rich lipoproteins and enhances lipolysis [18,19,20,21]. In addition to its role in regulating LPL activity, ApoC-III inhibits apolipoprotein E (ApoE)-mediated binding to LDL receptors, thereby limiting receptor-mediated hepatic clearance of triglyceride-rich lipoprotein remnants [21,22,23].

Other pathways contributing to hepatic clearance of triglyceride-rich lipoprotein remnants include interactions with heparan sulfate proteoglycans (HSPGs) and the LDL receptor-related protein (LRP), which act as co-receptors facilitating uptake [24]. Defective ApoE binding to these pathways leads to the accumulation of remnant particles and contributes to disorders such as familial dysbetalipoproteinemia (Type III hyperlipoproteinemia). Therefore, inhibition of ApoC-III function represents a therapeutic target in the management of hypertriglyceridemia and LPL-related genetic disorders [23,25].

The activity of LPL is also regulated by angiopoietin-like proteins (ANGPTLs), particularly ANGPTL3, ANGPTL4, and ANGPTL8. ANGPTL4 is induced during fasting and is highly expressed in adipose tissue, where it suppresses local LPL activity via paracrine and autocrine mechanisms to reduce triglyceride clearance and limit lipid uptake into adipocytes [26]. Under fed conditions, ANGPTL3 and ANGPTL8 are expressed in the liver, forming a complex interaction that inhibits LPL activity in oxidative tissues such as the heart and skeletal muscle [26,27]. These ANGPTL proteins serve as critical regulators of triglyceride metabolism [23]. Pharmacologic targeting of ANGPTLs represents a novel therapeutic approach for triglyceride-lowering, addressing residual cardiovascular risk and other triglyceride-related comorbidities [28].

## 3. Clinical Relevance of Remnant Lipoproteins in Atherosclerotic Cardiovascular Disease (ASCVD)

Triglyceride-rich lipoprotein remnants are well-established contributors to ASCVD [4,29,30,31]. These small remnant particles readily penetrate the arterial intima, where they are retained in the subendothelial space and contribute to endothelial dysfunction, inflammation, and the release of lipolysis-derived bioactive lipids. These processes promote monocyte adhesion and facilitate uptake of remnant lipoproteins by macrophages, leading to foam cell development and plaque progression [4,32]. Moreover, hypertriglyceridemia frequently coexists with metabolic abnormalities such as obesity, uncontrolled diabetes, and hepatic steatosis, all of which further increase ASCVD risk [32]. Recent cohort studies involving 93,461 individuals with up to 15 years of follow-up (2003 to 2015) demonstrated that ApoB-containing lipoproteins predict both peripheral artery disease (PAD) and coronary artery disease (CAD). However, remnant cholesterol was associated with a greater proportion of incident PAD events (73%; 95% CI, 32 to 100%) compared to LDL cholesterol (8%; 95% CI, 0 to 46%) [29].

## 4. Principles of Management: Primary vs. Secondary Hypertriglyceridemia

Managing hypertriglyceridemia requires careful identification of the underlying etiology. The biochemical and regulatory defects that characterize hereditary chylomicronemia syndromes differ markedly from those driving secondary hypertriglyceridemia [3,12]. These differences determine the appropriate pharmacologic approach and the clinical targets and outcomes that guide management. This etiologic distinction also determines treatment goals, which differ depending on whether triglyceride lowering is needed to prevent pancreatitis or to address residual ASCVD risk [3,32]. For these reasons, a comprehensive evaluation is essential. This includes a detailed review of medical and pharmacologic history, a careful physical examination, and targeted laboratory testing to assess glycemic control, kidney and liver function, thyroid status, and other potential contributors [2,3].

The majority of individuals with elevated triglyceride levels (>90%) develop this condition as a result of secondary contributing factors, multifactorial genetic susceptibility, or a combination of both [33]. These forms of hypertriglyceridemia generally respond well to lifestyle modification, management of underlying secondary contributors, and currently available triglyceride-lowering therapy, such as fibrates [3]. However, when the response to standard therapy is minimal, particularly in the presence of markedly elevated triglyceride levels (typically > 1000 mg/dL) and in the absence of identifiable secondary contributing factors, consideration of an underlying primary genetic disorder such as familial chylomicronemia syndrome (FCS) increases substantially. In this context, genetic testing is advised to confirm the diagnosis of FCS and guide targeted management strategies [12].

## 5. Current Therapies for the Management of Hypertriglyceridemia

Current pharmacologic therapies for hypertriglyceridemia provide variable triglyceride-lowering effects. Table 3 summarizes lipid-lowering therapies with established effects on triglyceride reduction.

### 5.1. Statins

Statins lower triglyceride levels through multiple mechanisms, including decreased hepatic VLDL synthesis due to reduced cholesterol availability, upregulation of LDL receptors enhancing clearance of VLDL remnants, and modest increases in lipolysis of triglyceride-rich lipoproteins [34]. Although their triglyceride-lowering effect is modest, statins are considered first-line treatment in individuals at intermediate or higher ASCVD risk who have mild to moderate hypertriglyceridemia (150–499 mg/dL), primarily because of their proven cardiovascular risk reduction benefits [2,3]. In these patients, statins typically provide a 10–30% dose-dependent reduction in triglyceride levels, in addition to lowering LDL cholesterol [2,3,34].

### 5.2. Omega-3 Fatty Acids

Omega-3 fatty acids are available in several forms, but those primarily responsible for reducing triglyceride levels are eicosapentaenoic acid (EPA) and docosahexaenoic acid (DHA) [35,36]. Although their exact mechanisms are not fully understood, omega-3 fatty acids are believed to produce their triglyceride-lowering effects through several pathways. These include inhibition of key enzymes responsible for triglyceride synthesis, such as diacylglycerol acyltransferase and phosphatidic acid phosphatase. Additionally, omega-3 fatty acids suppress the expression and activity of sterol regulatory element-binding protein 1c (SREBP-1c), a transcription factor that regulates lipogenesis. They also promote the breakdown of ApoB within hepatic cells, thereby reducing VLDL assembly and secretion. Furthermore, omega-3 fatty acids may enhance triglyceride clearance by increasing LPL activity and decreasing intrahepatic fatty acid stores that serve as precursors for triglyceride production [36]. EPA has been associated with more favorable cardiovascular benefits compared to DHA, potentially due to its ability to lower triglyceride levels without increasing LDL cholesterol, preserve membrane structure, reduce lipid oxidation, and modulate inflammatory pathways involved in atherosclerotic plaque development [37,38].

Prescription omega-3 fatty acids (EPA and DHA) can reduce triglyceride concentrations by approximately 20% to 60%, depending on the baseline triglyceride level and the dose administered [36]. Greater reductions are typically observed in patients with higher baseline triglyceride levels (≥500 mg/dL) and when doses of 3 to 4 g per day are used [36,39]. Additional factors, such as concomitant statin therapy and individual variability in lipoprotein metabolism, also influence the treatment response [36].

### 5.3. Fibrates

Fibrates exert their triglyceride-lowering effects primarily through activation of peroxisome proliferator-activated receptor-alpha (PPAR-α). This activation stimulates LPL expression and promotes the hydrolysis and clearance of triglyceride-rich lipoproteins. Fibrates are believed to downregulate hepatic production of ApoC-III and to potentially stimulate fatty acid β-oxidation, which limits substrate availability for triglyceride synthesis and reduces VLDL secretion [40]. In clinical use, fibrates generally lower plasma triglyceride concentrations by about 30% to 50%, with the magnitude of reduction depending on the baseline triglyceride level [2,3,34].

### 5.4. Niacin

Niacin lowers triglyceride levels by primarily inhibiting diacylglycerol acyltransferase-2 (DGAT2) in the liver, reducing triglyceride synthesis and VLDL assembly, which in turn decreases circulating triglycerides and LDL concentrations. In adipose tissue, niacin activates the GPR109A receptor, transiently suppressing lipolysis and free fatty acid release [41]. Therapeutic doses typically reduce plasma triglycerides by 20–50%, depending on baseline levels [2,3,34]. However, niacin is not routinely used for triglyceride lowering or ASCVD risk reduction due to limited evidence of cardiovascular benefit and frequent tolerability issues [3]. The most important safety considerations include flushing, which is common and often limits adherence, and dose-related hepatotoxicity, especially with sustained-release formulations. Niacin may also modestly worsen glycemic control [42].

**Table 3:** Comparative Overview of Currently Available Triglyceride-Lowering Therapies [3,8,23,36,40,41].

Therapy	Primary Target/Mechanism	TG Reduction
Statins	Inhibition of HMG-CoA reductase, leading to reduced hepatic VLDL production and increased clearance of triglyceride-rich lipoprotein remnants	~10–30%
Omega-3 fatty acids (EPA/DHA)	Exact mechanism is not fully understood; proposed to reduce hepatic triglyceride synthesis and VLDL secretion through suppression of lipogenesis and ApoB availability	~20–60%
Fibrates	Activation of PPAR-α, resulting in increased lipoprotein lipase activity and reduced ApoC-III expression, enhances triglyceride-rich lipoprotein clearance	~30–50%
Niacin	Reduction of hepatic triglyceride synthesis and VLDL production via inhibition of DGAT2 and decreased free fatty acid flux from adipose tissue	~20–50%
Other lipid-lowering agents, including ezetimibe, PCSK9 inhibitors, and bempedoic acid, may also produce modest reductions in triglyceride levels; however, their primary therapeutic role is in LDL cholesterol lowering.		

## 6. Emerging Therapies for the Management of Hypertriglyceridemia

**Figure 1:**
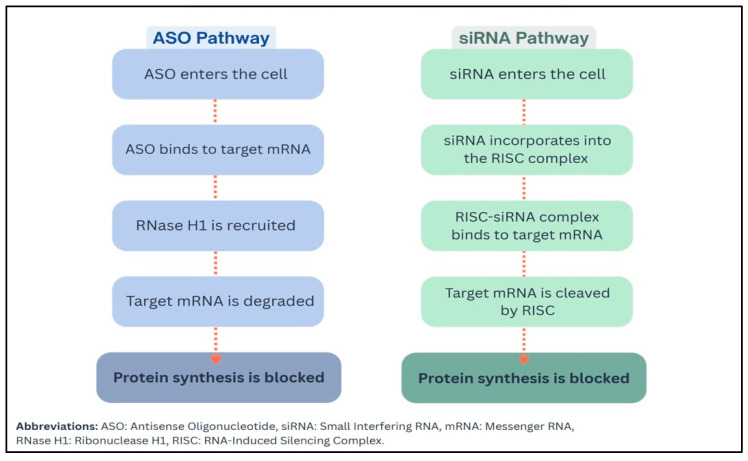
Simplified Mechanisms of Action of ASO and siRNA Therapies Targeting mRNA.

Emerging therapies utilizing antisense oligonucleotides (ASOs) and small interfering RNA (siRNA) have demonstrated substantial effects in lowering triglyceride levels (Table 4) [21,23,43,44,45]. These gene-silencing technologies exert their mechanism of action by selectively degrading messenger RNA transcripts that encode proteins critical to triglyceride metabolism, which results in reduced hepatic production and enhanced clearance of triglyceride-rich lipoproteins (Figure 1) [45]. ASOs and siRNA therapies primarily target proteins such as ApoC-III, ANGPTL3, ANGPTL4, and the ANGPTL3/8 complex [23,44,46]. Other investigational strategies include recombinant fibroblast growth factor 21 (FGF-21) analogues, which act through endocrine pathways to improve triglyceride metabolism [23,43,44]. Although most of these therapies remain under clinical investigation, a few agents have received regulatory approval for specific indications, including FCS and homozygous familial hypercholesterolemia (HoFH) [23,47]. Several of these management approaches are also being evaluated for potential benefits beyond severe hypertriglyceridemia, such as the prevention of acute pancreatitis, reduction of ASCVD risk in mild-to-moderate hypertriglyceridemia, and treatment of metabolic dysfunction-associated steatohepatitis (MASH) [43,48].

### 6.1. Apolipoprotein C-III (ApoC-III) Inhibitors

#### 6.1.1. Volanesorsen (Antisense Oligonucleotide Therapy)

Volanesorsen was the first agent developed to inhibit ApoC-III using the ASO approach and demonstrated substantial triglyceride reductions in randomized clinical trials [44,49,50,51]. In the phase 3 COMPASS trial, which enrolled patients with multifactorial chylomicronemia syndrome (MCS), treatment with volanesorsen at 300 mg subcutaneously once weekly resulted in a mean reduction in plasma triglyceride concentration of 71.2% (95% CI, −79.3 to −63.2; *p* < 0.0001) at 3 months, compared with 0.9% (95% CI, −13.9 to 12.2) in the placebo group [51]. In the phase 3 APPROACH trial of patients with FCS, the same 300 mg weekly regimen of volanesorsen achieved a mean triglyceride reduction of 77% at 3 months (*p* < 0.001), whereas patients receiving placebo experienced an 18% increase [49]. In the long-term open-label extension study, volanesorsen maintained triglyceride-lowering effects over 24 months in patients with FCS, with mean reductions ranging from 48% to 55% in those from the APPROACH trial, 42% to 66% among patients previously treated in COMPASS, and 46% to 60% in treatment-naive participants [50].

Thrombocytopenia was the principal safety concern associated with the use of volanesorsen. In the APPROACH trial, 76% of treated patients (25 of 33) experienced declines in platelet counts below 140,000/µL, and nearly half (48%; 16 of 33) had counts fall under 100,000/µL. Grade 4 thrombocytopenia with platelet counts below 25,000/µL occurred in 6% of patients (2 of 33) and led to treatment discontinuation and use of immunosuppressive therapy [49]. Long-term extension data confirmed that thrombocytopenia remained frequent, with confirmed platelet counts below 100,000/µL observed in approximately 50% of patients (33 of 68), resulting in 15% treatment discontinuation [50]. Although volanesorsen demonstrates robust triglyceride-lowering efficacy in patients with FCS and MCS, concerns primarily related to thrombocytopenia prevented its approval by the United States Food and Drug Administration (FDA). However, the European Medicines Agency (EMA) approved its use in Europe [43,44].

#### 6.1.2. Olezarsen (Antisense Oligonucleotide Therapy)

Olezarsen is the first ApoC-III inhibitor approved by the United States FDA for the management of hypertriglyceridemia in adults with FCS as an adjunct to dietary measures [52]. The primary difference between olezarsen and volanesorsen is the incorporation of an N-acetylgalactosamine (GalNAc) moiety, which enhances hepatic selectivity and uptake through high-affinity binding to the asialoglycoprotein receptor. This structural modification has been shown to reduce systemic adverse effects and improve dosing convenience and frequency while maintaining triglyceride-lowering efficacy relatively comparable to that of volanesorsen [53]. This pharmacologic advancement is also reflected in dosing strategies, as olezarsen achieves therapeutic effects with substantially lower and less frequent dosing regimens, typically 10 to 80 mg administered subcutaneously once monthly, whereas volanesorsen requires a fixed 300 mg subcutaneous injection administered on a weekly basis [43,44].

In the phase 3 Balance trial, 66 patients with genetically confirmed FCS and severe hypertriglyceridemia (mean baseline triglyceride level approximately 2630 mg/dL) were randomized to receive olezarsen 80 mg, olezarsen 50 mg, or placebo every four weeks for 49 weeks. The primary efficacy endpoint was the percent change in fasting triglyceride levels at six months. Olezarsen 80 mg resulted in a statistically significant placebo-corrected mean triglyceride reduction of 43.5% (95% CI, −69.1 to −17.9; *p* < 0.001). The 50 mg dose did not achieve statistical significance (−22.4% compared to placebo; 95% CI, −47.2 to +2.5; *p* = 0.08) [54]. The phase 2b Bridge-TIMI 73a randomized clinical trial enrolled a broader population and used the same primary endpoint as the Balance trial. Specifically, it included 154 patients with moderate hypertriglyceridemia or severe hypertriglyceridemia combined with elevated cardiovascular risk. Monthly administration of olezarsen at doses of 50 mg and 80 mg resulted in placebo-corrected triglyceride reductions of 49.3 percentage points (95% CI, −58.6 to −40.0) and 53.1 percentage points (95% CI, −61.5 to −44.6), respectively (*p* < 0.001 for both comparisons) [55]. In a prior phase 2 randomized dose-ranging study of 114 patients with moderate hypertriglyceridemia and either established cardiovascular disease or high cardiovascular risk, olezarsen administered at 10 mg every four weeks, 15 mg every two weeks, 10 mg weekly, or 50 mg every four weeks resulted in placebo-corrected mean triglyceride reductions of 23% (95% CI, −34% to −10%), 56% (95% CI, −62% to −49%), 60% (95% CI, −66% to −54%), and 60% (95% CI, −65% to −53%), respectively (*p* values ranged from 0.0042 to <0.0001) [56].

Olezarsen demonstrated an overall favorable safety profile across clinical trials. The most frequent adverse events were mild injection-site reactions, occurring in approximately 4–20% of patients, and transient flu-like symptoms occurred in 5–17%. Unlike volanesorsen, no clinically significant thrombocytopenia was observed, with platelet counts remaining above 100,000/mm^3^ in all participants [54,55,56]. Serious adverse events were infrequent and occurred at rates comparable to placebo [54,55]. In fact, olezarsen was associated with a reduction in episodes of acute pancreatitis compared to placebo in patients with a history of acute pancreatitis. In the Balance trial, which included patients with FCS, only 1 event occurred in each olezarsen group compared to 11 events in the placebo group [54]. These findings are significant because patients with FCS experience frequent and severe episodes of acute pancreatitis, and olezarsen offers a promising strategy to reduce this risk.

#### 6.1.3. Plozasiran (Small Interfering RNA Therapy)

Plozasiran, formerly known as ARO-APOC3, is another investigational agent that targets ApoC-III. The main distinction from volanesorsen and olezarsen is that its therapeutic effect is mediated through an siRNA mechanism. Similar to olezarsen, it has a GalNAc moiety that improves therapeutic efficacy and overall safety [44].

In the phase 3 PALISADE trial, 75 patients with persistent chylomicronemia and a median triglyceride level of 2044 mg/dL were enrolled. Among these participants, 44 had genetically confirmed FCS, and approximately 90% had a history of acute pancreatitis. After 10 months of treatment, median fasting triglyceride levels decreased by 80%, 78%, and 17% in the 25-mg group, the 50-mg group, and the placebo group, respectively (*p* < 0.001) [57]. Additionally, plozasiran resulted in substantial mean reductions in ApoC-III levels of 93% and 96% in the 25-mg and 50-mg groups, respectively, which exceeded the reductions reported with olezarsen (73.7% at 6 months) [54,57]. This difference may reflect the more profound target suppression achieved by siRNA within the nucleus compared to cytoplasmic ASO mechanism. The SHASTA-2 randomized phase 2b study evaluated plozasiran in 229 adults with severe hypertriglyceridemia (fasting triglycerides ≥500 mg/dL). At 24 weeks, plozasiran achieved placebo-corrected mean reductions in triglyceride levels of 53.1% (95% CI, −68.1% to −38.0%) in the 25 mg group and 57.0% (95% CI, −71.9% to −42.1%) in the 50 mg group [58]. The MUIR trial is another phase 2 trial that enrolled 353 patients with mixed hyperlipidemia, defined as fasting triglyceride levels between 150 and 499 mg/dL in combination with either LDL cholesterol ≥70 mg/dL or non-HDL cholesterol ≥100 mg/dL. In this study, plozasiran administered quarterly or semiannually produced significant placebo-corrected reductions in triglyceride levels at 24 weeks, ranging from −49.8% to −62.4% across dosing regimens (*p* < 0.001 for all dosing groups) [59].

Plozasiran demonstrated an overall favorable safety profile across clinical trials. The frequency of adverse events was similar between the plozasiran and placebo groups, with most events classified as mild to moderate in severity [57,58,59]. In the PALISADE trial, transient increases in alanine aminotransferase were reported in 23–46% of patients in the 25 mg and 50 mg dose groups, respectively, but did not exceed three times the upper limit of normal. Additionally, modest worsening of glycemic control occurred in 21–23% of patients receiving plozasiran compared with 8% in the placebo group. Platelet counts remained stable across all treatment arms. Consistent with observations from the BALANCE trial, a lower frequency of acute pancreatitis was documented in the PALISADE study, with 2 episodes occurring in the pooled plozasiran group compared with 7 events in the placebo group [57].

### 6.2. Angiopoietin-like Protein 3 (ANGPTL3) Inhibitors

#### 6.2.1. Evinacumab (Monoclonal Antibody)

Evinacumab binds to ANGPTL3 and inhibits its activity, resulting in the removal of the suppression of LPL and endothelial lipase and enhancing the clearance of triglyceride-rich lipoproteins and other atherogenic particles [44]. This agent has been approved by both the FDA and EMA for the treatment of HoFH [43]. Its primary lipid-lowering effects resulted in sustained LDL cholesterol reductions of approximately 40–55% in patients with refractory hypercholesterolemia and HoFH [60]. However, its efficacy in reducing triglyceride levels appears to depend on the presence of functional LPL activity, and therefore, patients with FCS due to bi-allelic LPL pathway mutations may not derive significant benefit [43]. This was demonstrated in a phase 2 randomized trial over 12 weeks, in which individuals with bi-allelic LPL mutations exhibited a median triglyceride reduction of −27.7% with evinacumab, compared to −22.9% with placebo (*p* = 0.95). In contrast, patients with heterozygous mutations or without LPL pathway mutations achieved significantly greater reductions of −64.8% (*p* = 0.008) and −81.7% (*p* = 0.04), respectively [61]. Further investigation is needed to clarify whether higher doses of evinacumab or other ANGPTL3 inhibitors could achieve more substantial triglyceride lowering in this population [43].

Evinacumab was generally well tolerated in patients with severe hypertriglyceridemia. The overall frequency of treatment-emergent adverse events was similar between evinacumab (71.4%) and placebo (68.8%). A total of 5 episodes of acute pancreatitis occurred during the double-blind treatment period, including 2 events in a single patient receiving evinacumab, which were attributed to persistently elevated triglyceride levels [61].

#### 6.2.2. Vupanorsen (Antisense Oligonucleotide Therapy)

Vupanorsen targets ANGPTL3 and was evaluated for the management of mild to moderate hypertriglyceridemia (150–500 mg/dL). However, its development was discontinued in January 2022 due to significant increases in hepatic fat fraction despite favorable effects on lipid parameters [44]. In the phase 2b TRANSLATE-TIMI 70 trial, vupanorsen significantly reduced triglycerides by 41–57% in statin-treated patients with mild to moderate hypertriglyceridemia (150–500 mg/dL). However, these lipid-lowering benefits were associated with dose-dependent increases in hepatic fat fraction of up to 76% at the highest doses, as well as higher rates of liver enzyme elevations and injection site reactions, ultimately leading to discontinuation of development [62]. In a secondary analysis of the TRANSLATE-TIMI 70 trial, vupanorsen was associated with dose-dependent absolute increases in hepatic fat fraction of up to 7.0% at the highest dose (*p* < 0.001) over 24 weeks. The underlying mechanism driving this increase in hepatic fat remains uncertain. One hypothesis is that it may represent an off-target effect unique to the ASO approach, as similar findings have not been observed with ANGPTL3 inhibition using monoclonal antibodies or siRNA. Alternatively, it may reflect a direct consequence of ANGPTL3 suppression on intracellular lipid storage [63].

Despite these safety concerns, vupanorsen demonstrated robust lipid-lowering efficacy targeting atherogenic lipoproteins. Another secondary analysis of the TRANSLATE-TIMI 70 trial showed that vupanorsen produced substantial placebo-adjusted percentage reductions in remnant cholesterol by up to 59% and VLDL cholesterol by up to 67% over 24 weeks, both of which are recognized contributors to residual ASCVD risk [64]. These findings suggest that ANGPTL3 inhibition may represent a promising strategy for targeting triglyceride-rich lipoproteins in ASCVD prevention, although further outcome trials are needed to establish clinical benefit.

#### 6.2.3. Zodasiran (Small Interfering RNA Therapy)

Zodasiran, formerly known as ARO-ANG3, targets ANGPTL3 and was evaluated for the management of mild to moderate hypertriglyceridemia (150–499 mg/dL) [44]. In the ARCHES-2 phase 2b trial, 204 patients with mixed hyperlipidemia and triglyceride levels between 150–499 mg/dL were randomized to receive zodasiran (50, 100, or 200 mg) or placebo. At 24 weeks, zodasiran produced significant, dose-dependent, placebo-adjusted reductions in triglycerides of −51, −57, and −63 percentage points for the 50, 100, and 200 mg doses, respectively (*p* < 0.001 for all dosing groups). ANGPTL3 levels decreased by −54% to −74% depending on the administered dose, and these reductions were associated with significant placebo-adjusted decreases in non-HDL cholesterol (up to −36%), ApoB (up to −22%), and LDL cholesterol (up to −20%) [65].

Zodasiran demonstrated an overall favorable safety profile in the ARCHES-2 trial. Unlike vupanorsen, zodasiran resulted in dose-dependent reductions in liver fat fraction, with the highest dose group showing a mean decrease of −28.4% at week 25 [65]. This reduction in hepatic fat is particularly relevant given earlier concerns about hepatic steatosis observed with vupanorsen [62,63,64]. Additionally, among patients with preexisting diabetes, there was a modest increase in glycated hemoglobin (HbA1c) over 24 weeks, with the mean rising from 7.4% at baseline to 7.7% in the 200 mg group, suggesting potential effects on glycemic control that warrant further investigation [65].

### 6.3. Fibroblast Growth Factor 21 (FGF-21)

FGF-21 is an endocrine peptide hormone primarily produced by the liver that exerts widespread metabolic effects via signaling pathways requiring the β-Klotho coreceptor [66]. Its role in metabolic regulation has generated increasing therapeutic interest across cardiometabolic disorders, including obesity, insulin resistance, fatty liver disease, and hypertriglyceridaemia [67,68,69]. However, endogenous FGF-21 exhibits limited circulating persistence, and modified FGF-21 analogues have therefore been developed to extend biological exposure and enable sustained receptor engagement [69]. In the context of lipid metabolism, pharmacologic FGF-21 analogs lower circulating nonesterified fatty acids, reduce hepatic de novo lipogenesis, and inhibit VLDL secretion. These mechanisms result in decreased hepatic triglyceride accumulation and improved clearance of triglyceride-rich lipoproteins. Additionally, FGF-21 enhances adiponectin production in adipose tissue, further promoting fatty acid oxidation and improving lipid profiles [66].

#### Pegozafermin (Fibroblast Growth Factor 21 Therapy)

Pegozafermin is a glycopegylated, long-acting analog representing a novel approach to managing hypertriglyceridemia and related metabolic disorders. In the phase 2b ENTRIGUE trial, pegozafermin was evaluated in 85 patients with severe hypertriglyceridemia and a median baseline triglyceride level of 622 mg/dL [70]. At week 8, all dosing regimens (9 mg, 18 mg, and 27 mg once weekly, or 36 mg every two weeks) achieved median reductions in triglyceride levels ranging from −57% to −63%, compared with −5% for placebo (*p* < 0.001 for all dosing comparisons). Additionally, patients receiving the 27 mg weekly dose experienced a least-squares mean reduction in liver fat content of −42.2%, compared with −8.3% for placebo (*p* = 0.012), suggesting that pegozafermin may also confer beneficial effects on hepatic steatosis. The therapy was generally well tolerated, with no treatment-related serious adverse events. Mild gastrointestinal symptoms and injection site reactions were the most frequently reported side effects, and no significant liver enzyme elevations or treatment-related deaths were observed. Although these results are encouraging, the ongoing phase 3 ENTRUST trial is designed to further evaluate its efficacy and safety in patients with triglyceride levels between 500 and 2000 mg/dL [71].

## 7. Clinical Considerations and Practical Perspectives

Emerging therapies targeting triglyceride metabolism offer promising new options for patients with hypertriglyceridemia. Despite the availability of established triglyceride-lowering agents, many individuals with severe or refractory disease often remain inadequately controlled [44]. ApoC-III inhibitors such as ASO olezarsen and the siRNA plozasiran have achieved triglyceride reductions of 50–80%. ANGPTL3 inhibitors, including the monoclonal antibody evinacumab and the siRNA zodasiran, have also shown significant triglyceride reductions of 30–80%. Additionally, FGF-21 analogs like pegozafermin have provided encouraging triglyceride-lowering effects, although this therapy is still in the early stages of development. These agents may also offer potential benefits for preventing acute pancreatitis, lowering ASCVD risk, and managing MASH; however, their use in these indications remains investigational, and their outcomes remain uncertain. Table 5 presents a detailed summary of the key characteristics of emerging triglyceride-lowering therapies, including ApoC-III inhibitors, ANGPTL3 inhibitors, and FGF-21 analogs. Moreover, early-phase trials are actively evaluating pharmacologic inhibitors targeting the ANGPTL3/8 complex and ANGPTL4 pathways, which hold promise for expanding the therapeutic landscape of triglyceride reduction and for addressing residual atherosclerotic cardiovascular risk [43].

Despite these therapeutic advances, it remains essential for both clinicians and patients to recognize that these emerging agents are not a substitute for comprehensive lifestyle modification. Regular physical activity and weight loss should be discussed with all patients with hypertriglyceridemia. Additionally, patients should be advised to establish a consistent dietary pattern that includes limiting total fat intake, reducing added sugars, and choosing complex carbohydrates over refined starches. Maintaining these lifestyle habits over the long term is essential to enhance the effectiveness of pharmacologic treatment and reduce overall cardiometabolic risk [11]. It is also important to maintain vigilance and assess for secondary causes of hypertriglyceridemia, such as uncontrolled diabetes, alcohol use, medications, and hypothyroidism [2,3,4,5,72].

While triglyceride reduction is an important therapeutic goal, clinicians should also assess the overall lipid profile, the risk of pancreatitis, and the atherosclerotic burden to guide comprehensive management decisions [73]. Additionally, since hypertriglyceridemia is often associated with metabolic derangements, including type 2 diabetes and obesity, it is recommended to evaluate the risks and benefits of using glucagon-like peptide-1 (GLP-1) receptor agonists in the treatment strategy [74,75].

Overall, these emerging therapies have demonstrated an acceptable safety profile, although some agents require more vigilant monitoring. For example, volanesorsen necessitates regular assessment of platelet counts due to a well-documented risk of thrombocytopenia [49,51]. siRNA-based treatments such as plozasiran and zodasiran are generally well tolerated but warrant periodic monitoring of liver enzymes and glycemic parameters [57,58,65]. FGF21 analogs like pegozafermin have been associated with increased gastrointestinal symptoms and occasional elevations in hepatic transaminases in early-phase studies; however, these findings remain preliminary and will require confirmation in larger trials [70]. Given the rarity of the diseases studied and the relatively small sample sizes of the available trials, real-world experience and longer-term data will be essential to provide a more comprehensive understanding of the safety and tolerability of these emerging therapies.

**Table 5:** Emerging triglyceride-lowering agents: mechanisms, indications, and key characteristics.

Agent	Therapeutic Modality	Potential Indications	Development Status	Dose Regimens	TG-Lowering Efficacy	Notable Safety Considerations
ApoC-III Inhibitors						
Volanesorsen	ASO	FCS, MCS, FPLD	EMA approved for FCS; FDA not approved	300 mg SC weekly [49,51,76]	−71% to −77% [49,51]	High rates of thrombocytopenia
Olezarsen	ASO	FCS, severe HTG, moderate HTG with ASCVD risk	FDA approved for FCS; Phase 3 ongoing: NCT05130450 NCT05185843 NCT05079919 NCT05552326	10–50 mg SC (dose-ranging study) [56] 80 mg SC q4w [54,55]	−23 to −60% [56] −22% to −43% [54] −49 to −53 pp [55]	Mild-Moderate injection reactions; no significant thrombocytopenia
Plozasiran	siRNA	Persistent chylomicronemia, severe/moderate HTG, mixed dyslipidemia	Investigational NCT06347016 NCT06347003 NCT05902598 NCT06347133	25–50 mg SC q12w [57] 10, 25, 50 mg SC q12w [58] 10, 25, 50 mg SC q12w; 50 mg q24w [59]	−78% to −80% [57] −53% to −57% [58] −49.8% to −62.4% [59]	Transient ALT elevations; mild worsening of glycemic control
ANGPTL3 Inhibitors						
Zodasiran	siRNA	Mixed hyperlipidemia (TG 150–499 mg/dL)	Investigational NCT07037771 NCT06712771	50, 100, 200 mg SC q12w [65]	−51 to −63 pp [65]	Modest HbA1c rise with highest dose; hepatic fat reduction
Evinacumab	Monoclonal antibody	Severe HTG HoFH	FDA approved for HoFH	15 mg/kg IV q4w [61]	−27% to −82% median reduction depending on LPL genotype [61]	Generally well tolerated; similar adverse event rates vs. placebo
Vupanorsen	ASO	Mild–moderate HTG, ASCVD prevention	Discontinued	60, 80, 120, 160 mg SC q2w; 80, 120, 160 mg SC q4w (dose-ranging) [62]	−41% to −57% [62]	~76% hepatic fat increase; ALT > 3× ULN in 44% at the highest dose
FGF-21 Analog						
Pegozafermin	FGF-21 analog	Severe HTG, MASH	Investigational NCT05852431	9, 18, 27 mg SC q1w; 36 mg SC q2w [70]	−57% to −63% median reduction [70]	Mild GI symptoms; liver fat reduction

Abbreviations: ALT = alanine aminotransferase; ASCVD = atherosclerotic cardiovascular disease; ASO = antisense oligonucleotide; FCS = familial chylomicronemia syndrome; FGF-21 = fibroblast growth factor 21; FPLD = familial partial lipodystrophy; GI = gastrointestinal; HoFH = homozygous familial hypercholesterolemia; HTG = hypertriglyceridemia; IV = intravenous; LPL = lipoprotein lipase; MASH = metabolic dysfunction-associated steatohepatitis; MCS = multifactorial chylomicronemia syndrome; pp = percentage points; q2w = every 2 weeks; q4w = every 4 weeks; q12w = every 12 weeks; q24w = every 24 weeks; SC = subcutaneous.

## 8. Conclusions

The clinical significance of hypertriglyceridemia is supported by a robust and expanding body of evidence linking it to residual cardiovascular risk and other adverse health outcomes. Current lipid-lowering therapies provide variable reductions in triglyceride levels; however, many offer only modest triglyceride lowering while primarily targeting LDL-C. Emerging therapies that target key regulators of triglyceride metabolism provide substantial triglyceride-lowering efficacy and represent a promising advancement, particularly for individuals with genetic or severe forms of hypertriglyceridemia who exhibit suboptimal response to currently available therapies. Nevertheless, as with any novel pharmacological therapies, long-term safety, cost-effectiveness, and impact on cardiovascular outcomes will ultimately determine their role in clinical practice.

## Figures and Tables

**Table 1:** Comparison of triglyceride classification thresholds by major societies.

ACC/AHA (2018) [2]	ACC Consensus (2021) [3]	EAS (2021) [4]	Endocrine Society (2012) [5]
Normal < 175 mg/dL	Normal < 150 mg/dL	Optimal < 100 mg/dL	Optimal < 150 mg/dL
Moderate 175–499 mg/dL	Moderate 150–499 mg/dL	Borderline 100–150 mg/dL	Mild 150–199 mg/dL
Severe ≥ 500 mg/dL	Severe ≥ 500 mg/dL	Moderately elevated 150–500 mg/dL	Moderate 200–999 mg/dL
		Severe 500–880 mg/dL	Severe 1000–1999 mg/dL
		Extreme > 880 mg/dL	Very severe ≥ 2000 mg/dL

Abbreviations: ACC: American College of Cardiology; AHA: American Heart Association; EAS: European Atherosclerosis Society.

**Table 2:** Recommended lifestyle interventions by triglyceride level [3,11,12].

Lifestyle Factor	Mild–Moderate (TG 150–500 mg/dL)	Moderate–Severe (TG 500–999 mg/dL)	Severe (TG ≥ 1000 mg/dL)
Dietary fat intake (Dietary patterns)	Adjust fat intake to 25–35% of total calories, prioritizing unsaturated fats.	Reduce fat intake to 20–25% of calories; further restriction (<15%) may be necessary in high-risk patients.	Restrict fat to <10–15% of calories (~10–20 g/day). Ensure adequate essential fatty acid intake.
Added sugars	Limit added sugars (including honey and syrups) to <6% of daily intake.	Strictly reduce added sugars to <5% of daily calories.	Eliminate added sugars from diet to reduce hepatic triglyceride production.
Alcohol	Minimize alcohol; if consumed, limit to 1 drink per day.	Avoid alcohol completely, due to high risk of severe hypertriglyceridemia.	Strictly avoid alcohol, as even small amounts can trigger pancreatitis.
Carbohydrates	Focus on complex carbs and fiber-rich foods while limiting refined carbohydrates.	Reduce refined carbohydrates; consider replacing with unsaturated fats if appropriate.	Emphasize complex carbohydrates (vegetables, legumes, whole grains); avoid refined starches.
Omega-3 fatty acids	Include ≥ 2 servings/week of fatty fish; prescription omega-3s may be considered if needed.	Maintain omega-3 intake; prescription products may be used adjunctively.	May be considered in some patients, though limited efficacy in FCS.
Physical activity	Engage in ≥150 min/week moderate aerobic exercise, or ≥75 min/week vigorous activity, or equivalent combination.		
Weight management	5–10% reduction in body weight.		
Monitoring	Monitor triglyceride response and adjust plan as needed.		Require regular monitoring In MCS, cautious fat liberalization if TG < 500 mg/dL In FCS, lifelong restriction
Nutrition counseling & specialist care	Assess for secondary causes (e.g., diabetes, medications); consider referral to a registered dietitian.	Strongly recommend referral to a dietitian experienced in hypertriglyceridemia management.	Referral to a dietitian is essential, especially for long-term management and prevention of nutrient deficiencies.

Abbreviations: FCS: familial chylomicronemia syndrome; MCS: multifactorial chylomicronemia syndrome; TG: triglycerides.

**Table 4:** Comparison of Antisense Oligonucleotide and Small Interfering RNA Gene-Silencing Approaches [21,43,44,45,46].

Characteristic	Antisense Oligonucleotides (ASOs)	Small Interfering RNA (siRNA)
Chemical structure	Single-stranded RNA	Double-stranded RNA
Primary site of action	Nucleus and cytoplasm	Cytoplasm
Mechanism of action	Binds target mRNA and induces RNase H-mediated degradation	Incorporated into RISC, enabling enzymatic cleavage of target mRNA
Examples of therapeutic agents	ApoC-III inhibitors (volanesorsen, olezarsen), ANGPTL3 inhibitors (vupanorsen; discontinued)	ApoC-III inhibitors (polzasiran), ANGPTL3 inhibitors (zodasiran)
Specificity	High target specificity	High target specificity
Immunogenicity	Potential for immune activation varies by chemical modifications	Potential for immune activation varies by chemical modifications
Pharmacokinetic profile	Tissue half-life: typically 2–4 weeks	Functional duration ~3–6 months
Administration	Subcutaneous injection	Subcutaneous injection
Dosing frequency	Monthly (some quarterly regimens in development)	Every 3–6 months
Efficacy	Approximately −44% to −94% reduction, depending on the therapeutic target and baseline triglyceride levels	Approximately −56% to −63% reduction, depending on the therapeutic target and baseline triglyceride levels
Delivery enhancement	GalNAc moiety conjugation enhances selective uptake by liver through high-affinity binding to the asialoglycoprotein receptor, improving delivery efficiency, allowing for reduced dosing and frequency, and consequently minimizing systemic exposure and associated adverse effects. Volanesorsen lacked this feature and therefore showed higher rates of systemic adverse effects compared to other agents.	
Note	Some agents approved (volanesorsen EU/UK; olezarsen FDA December 2024)	In clinical development

Abbreviations: ANGPTL3—angiopoietin-like protein 3; ApoC-III—apolipoprotein C-III; ASO—antisense oligonucleotide; EU—European Union; GalNAc—N-acetylgalactosamine; mRNA—messenger RNA; RISC—RNA-induced silencing complex; siRNA—small interfering RNA; UK—United Kingdom.

## Data Availability

No new data were created or analyzed in this study. Data sharing is not applicable to this article.

## Abbreviations

The following abbreviations are used in this manuscript:

ACC	American College of Cardiology
AHA	American Heart Association
ALT	Alanine aminotransferase
ANGPTL3	Angiopoietin-like protein 3
ANGPTL4	Angiopoietin-like protein 4
ANGPTL8	Angiopoietin-like protein 8
ApoA5	Apolipoprotein A5
ApoB	Apolipoprotein B
ApoB48	Apolipoprotein B 48
ApoB100	Apolipoprotein B 100
ApoC-II	Apolipoprotein C-II
ApoC-III	Apolipoprotein C-III
ApoE	Apolipoprotein E
ASCVD	Atherosclerotic cardiovascular disease
ASO	Antisense oligonucleotide
CAD	Coronary artery disease
CI	Confidence interval
DGAT2	Diacylglycerol acyltransferase 2
DHA	Docosahexaenoic acid
EAS	European Atherosclerosis Society
EMA	European Medicines Agency
EPA	Eicosapentaenoic acid
EU	European Union
FCS	Familial chylomicronemia syndrome
FDA	Food and Drug Administration
FGF-21	Fibroblast growth factor 21
FPLD	Familial partial lipodystrophy
GalNAc	N-Acetylgalactosamine
GLP-1	Glucagon-like peptide-1
HbA1c	Glycated hemoglobin
HoFH	Homozygous familial hypercholesterolemia
HSPGs	Heparan sulfate proteoglycans
HTG	Hypertriglyceridemia
IV	Intravenous
LDL	Low-density lipoprotein
LPL	Lipoprotein lipase
LRP	LDL receptor-related protein
MASH	Metabolic dysfunction-associated steatohepatitis
MCS	Multifactorial chylomicronemia syndrome
mRNA	Messenger RNA
MTTP	Microsomal triglyceride transfer protein
NHANES	National Health and Nutrition Examination Survey
PAD	Peripheral artery disease
PPAR-α	Peroxisome proliferator-activated receptor alpha
pp	Percentage points
q2w	Every 2 weeks
q4w	Every 4 weeks
q12w	Every 12 weeks
q24w	Every 24 weeks
RISC	RNA-induced silencing complex
SC	Subcutaneous
siRNA	Small interfering RNA
SREBP-1c	Sterol regulatory element-binding protein 1c
TG	Triglycerides
UK	United Kingdom
VLDL	Very-low-density lipoprotein

## References

[1] Saadatagah S., Naderian M., Larouche M., Gaudet D., Kullo I.J., Ballantyne C.M. (2025). Epidemiology and longitudinal course of chylomicronemia: Insights from NHANES and a large health care system. J. Clin. Lipidol..

[2] Grundy S.M., Stone N.J., Bailey A.L., Beam C., Birtcher K.K., Blumenthal R.S., Braun L.T., De Ferranti S., Faiella-Tommasino J., Forman D.E. (2019). 2018 AHA/ACC/AACVPR/AAPA/ABC/ACPM/ADA/AGS/APhA/ASPC/NLA/PCNA Guideline on the Management of Blood Cholesterol: A Report of the American College of Cardiology/American Heart Association Task Force on Clinical Practice Guidelines. Circulation.

[3] Virani S.S., Morris P.B., Agarwala A., Ballantyne C.M., Birtcher K.K., Kris-Etherton P.M., Ladden-Stirling A.B., Miller M., Orringer C.E., Stone N.J. (2021). 2021 ACC Expert Consensus Decision Pathway on the Management of ASCVD Risk Reduction in Patients With Persistent Hypertriglyceridemia: A Report of the American College of Cardiology Solution Set Oversight Committee. J. Am. Coll. Cardiol..

[4] Ginsberg H.N., Packard C.J., Chapman M.J., Borén J., Aguilar-Salinas C.A., Averna M., Ference B.A., Gaudet D., Hegele R.A., Kersten S. (2021). Triglyceride-rich lipoproteins and their remnants: Metabolic insights, role in atherosclerotic cardiovascular disease, and emerging therapeutic strategies-a consensus statement from the European Atherosclerosis Society. Eur. Heart J..

[5] Berglund L., Brunzell J.D., Goldberg A.C., Goldberg I.J., Sacks F., Murad M.H., Stalenhoef A.F.H., Endocrine Society (2012). Evaluation and treatment of hypertriglyceridemia: An Endocrine Society clinical practice guideline. J. Clin. Endocrinol. Metab..

[6] Adiels M., Olofsson S.-O., Taskinen M.-R., Borén J. (2008). Overproduction of very low-density lipoproteins is the hallmark of the dyslipidemia in the metabolic syndrome. Arterioscler. Thromb. Vasc. Biol..

[7] Castañer O., Pintó X., Subirana I., Amor A.J., Ros E., Hernáez Á., Martínez-González M.Á., Corella D., Salas-Salvadó J., Estruch R. (2020). Remnant Cholesterol, Not LDL Cholesterol, Is Associated with Incident Cardiovascular Disease. J. Am. Coll. Cardiol..

[8] Alhomoud I.S., Talasaz A., Mehta A., Kelly M.S., Sisson E.M., Bucheit J.D., Brown R., Dixon D.L. (2023). Role of lipoprotein(a) in atherosclerotic cardiovascular disease: A review of current and emerging therapies. Pharmacother. J. Hum. Pharmacol. Drug Ther..

[9] Van Gaal L.F., Mertens I.L., Ballaux D. (2005). What is the relationship between risk factor reduction and degree of weight loss?. Eur. Heart J. Suppl..

[10] Wood P.D., Stefanick M.L., Williams P.T., Haskell W.L. (1991). The Effects on Plasma Lipoproteins of a Prudent Weight-Reducing Diet, with or without Exercise, in Overweight Men and Women. N. Engl. J. Med..

[11] Kirkpatrick C.F., Sikand G., Petersen K.S., Anderson C.A.M., Aspry K.E., Bolick J.P., Kris-Etherton P.M., Maki K.C. (2023). Nutrition interventions for adults with dyslipidemia: A Clinical Perspective from the National Lipid Association. J. Clin. Lipidol..

[12] Javed F., Hegele R.A., Garg A., Patni N., Gaudet D., Williams L., Khan M., Li Q., Ahmad Z. (2025). Familial chylomicronemia syndrome: An expert clinical review from the National Lipid Association. J. Clin. Lipidol..

[13] Laufs U., Parhofer K.G., Ginsberg H.N., Hegele R.A. (2020). Clinical review on triglycerides. Eur. Heart J..

[14] Lo C.C., Coschigano K.T. (2020). ApoB48 as an Efficient Regulator of Intestinal Lipid Transport. Front. Physiol..

[15] Wit M., Trujillo-Viera J., Strohmeyer A., Klingenspor M., Hankir M., Sumara G. (2022). When fat meets the gut-focus on intestinal lipid handling in metabolic health and disease. EMBO Mol. Med..

[16] van Zwol W., van de Sluis B., Ginsberg H.N., Kuivenhoven J.A. (2024). VLDL Biogenesis and Secretion: It Takes a Village. Circ. Res..

[17] Bredefeld C., Hussain M.M., Averna M., Black D.D., Brin M.F., Burnett J.R., Charrière S., Cuerq C., Davidson N.O., Deckelbaum R.J. (2022). Guidance for the diagnosis and treatment of hypolipidemia disorders. J. Clin. Lipidol..

[18] Watts G.F. (2024). Shooting the Messenger to Treat Hypertriglyceridemia. N. Engl. J. Med..

[19] Yang Y., Konrad R.J., Ploug M., Young S.G. (2024). APOA5 deficiency causes hypertriglyceridemia by reducing amounts of lipoprotein lipase in capillaries. J. Lipid Res..

[20] Wen Y., Chen Y.Q., Konrad R.J. (2022). The Regulation of Triacylglycerol Metabolism and Lipoprotein Lipase Activity. Adv. Biol..

[21] Tomlinson B., Wu Q.-Y., Zhong Y.-M., Li Y.-H. (2024). Advances in Dyslipidaemia Treatments: Focusing on ApoC3 and ANGPTL3 Inhibitors. J. Lipid Atheroscler..

[22] Chebli J., Larouche M., Gaudet D. (2024). APOC3 siRNA and ASO therapy for dyslipidemia. Curr. Opin. Endocrinol. Diabetes Obes..

[23] Zimodro J.M., Rizzo M., Gouni-Berthold I. (2025). Current and Emerging Treatment Options for Hypertriglyceridemia: State-of-the-Art Review. Pharmaceuticals.

[24] Mahley R.W., Huang Y. (2007). Atherogenic remnant lipoproteins: Role for proteoglycans in trapping, transferring, and internalizing. J. Clin. Investig..

[25] Brinton E.A., Eckel R.H., Gaudet D., Ballantyne C.M., Baker B.F., Ginsberg H.N., Witztum J.L. (2025). Familial chylomicronemia syndrome and treatments to target hepatic APOC3 mRNA. Atherosclerosis.

[26] Sylvers-Davie K.L., Davies B.S.J. (2021). Regulation of lipoprotein metabolism by ANGPTL3, ANGPTL4, and ANGPTL8. Am. J. Physiol. Endocrinol. Metab..

[27] Jin N., Matter W.F., Michael L.F., Qian Y., Gheyi T., Cano L., Perez C., Lafuente C., Broughton H.B., Espada A. (2021). The Angiopoietin-Like Protein 3 and 8 Complex Interacts with Lipoprotein Lipase and Induces LPL Cleavage. ACS Chem. Biol..

[28] Vahdat-Lasemi F., Farhoudi L., Hosseinikhah S.M., Santos R.D., Sahebkar A. (2025). Angiopoietin-like protein inhibitors: Promising agents for the treatment of familial hypercholesterolemia and atherogenic dyslipidemia. Atherosclerosis.

[29] Wadström B.N., Pedersen K.M., Wulff A.B., Nordestgaard B.G. (2024). Remnant Cholesterol, Not LDL Cholesterol, Explains Peripheral Artery Disease Risk Conferred by apoB: A Cohort Study. Arterioscler. Thromb. Vasc. Biol..

[30] Nordestgaard B.G. (2016). Triglyceride-Rich Lipoproteins and Atherosclerotic Cardiovascular Disease: New Insights From Epidemiology, Genetics, and Biology. Circ. Res..

[31] Björnson E., Adiels M., Taskinen M.-R., Burgess S., Rawshani A., Borén J., Packard C.J. (2023). Triglyceride-rich lipoprotein remnants, low-density lipoproteins, and risk of coronary heart disease: A UK Biobank study. Eur. Heart J..

[32] Watts G.F., Ooi E.M.M., Chan D.C. (2013). Demystifying the management of hypertriglyceridaemia. Nat. Rev. Cardiol..

[33] Dron J.S., Wang J., Cao H., McIntyre A.D., Iacocca M.A., Menard J.R., Movsesyan I., Malloy M.J., Pullinger C.R., Kane J.P. (2019). Severe hypertriglyceridemia is primarily polygenic. J. Clin. Lipidol..

[34] Miller M., Stone N.J., Ballantyne C., Bittner V., Criqui M.H., Ginsberg H.N., Goldberg A.C., Howard W.J., Jacobson M.S., Kris-Etherton P.M. (2011). Triglycerides and Cardiovascular Disease: A Scientific Statement From the American Heart Association. Circulation.

[35] Balk E.M., Adams G.P., Langberg V., Halladay C., Chung M., Lin L., Robertson S., Yip A., Steele D., Smith B.T. (2016). Omega-3 Fatty Acids and Cardiovascular Disease: An Updated Systematic Review. Evid. Rep. Technol. Assess. (Full Rep.).

[36] Skulas-Ray A.C., Wilson P.W.F., Harris W.S., Brinton E.A., Kris-Etherton P.M., Richter C.K., Jacobson T.A., Engler M.B., Miller M., Robinson J.G. (2019). Omega-3 Fatty Acids for the Management of Hypertriglyceridemia: A Science Advisory From the American Heart Association. Circulation.

[37] Mason R.P., Libby P., Bhatt D.L. (2020). Emerging Mechanisms of Cardiovascular Protection for the Omega-3 Fatty Acid Eicosapentaenoic Acid. Arterioscler. Thromb. Vasc. Biol..

[38] Bhatt D.L., Steg P.G., Miller M., Brinton E.A., Jacobson T.A., Ketchum S.B., Doyle R.T., Juliano R.A., Jiao L., Granowitz C. (2019). Cardiovascular Risk Reduction with Icosapent Ethyl for Hypertriglyceridemia. N. Engl. J. Med..

[39] Bays H.E., Ballantyne C.M., Kastelein J.J., Isaacsohn J.L., Braeckman R.A., Soni P.N. (2011). Eicosapentaenoic Acid Ethyl Ester (AMR101) Therapy in Patients with Very High Triglyceride Levels (from the Multi-center, plAcebo-controlled, Randomized, double-blINd, 12-week study with an open-label Extension [MARINE] Trial). Am. J. Cardiol..

[40] Staels B., Dallongeville J., Auwerx J., Schoonjans K., Leitersdorf E., Fruchart J.C. (1998). Mechanism of action of fibrates on lipid and lipoprotein metabolism. Circulation.

[41] Kamanna V.S., Kashyap M.L. (2008). Mechanism of action of niacin. Am. J. Cardiol..

[42] Guyton J.R., Bays H.E. (2007). Safety considerations with niacin therapy. Am. J. Cardiol..

[43] Nordestgaard A.T., Tybjærg-Hansen A., Mansbach H., Kersten S., Nordestgaard B.G., Rosenson R.S. (2025). Target Populations for Novel Triglyceride-Lowering Therapies. J. Am. Coll. Cardiol..

[44] Gouni-Berthold I., Schwarz J., Berthold H.K. (2023). Updates in Drug Treatment of Severe Hypertriglyceridemia. Curr. Atheroscler. Rep..

[45] Springer A.D., Dowdy S.F. (2018). GalNAc-siRNA Conjugates: Leading the Way for Delivery of RNAi Therapeutics. Nucleic. Acid. Ther..

[46] Nordestgaard B.G., Nicholls S.J., Langsted A., Ray K.K., Tybjærg-Hansen A. (2018). Advances in lipid-lowering therapy through gene-silencing technologies. Nat. Rev. Cardiol..

[47] Alhomoud I.S. (2025). Emerging therapies targeting lipoprotein(a): The next frontier in cardiovascular risk reduction. Front. Med..

[48] Bornfeldt K.E. (2024). Apolipoprotein C3: Form begets function. J. Lipid. Res..

[49] Witztum J.L., Gaudet D., Freedman S.D., Alexander V.J., Digenio A., Williams K.R., Yang Q., Hughes S.G., Geary R.S., Arca M. (2019). Volanesorsen and Triglyceride Levels in Familial Chylomicronemia Syndrome. N. Engl. J. Med..

[50] Witztum J.L., Gaudet D., Arca M., Jones A., Soran H., Gouni-Berthold I., Stroes E.S.G., Alexander V.J., Jones R., Watts L. (2023). Volanesorsen and triglyceride levels in familial chylomicronemia syndrome: Long-term efficacy and safety data from patients in an open-label extension trial. J. Clin. Lipidol..

[51] Gouni-Berthold I., Alexander V.J., Yang Q., Hurh E., Steinhagen-Thiessen E., Moriarty P.M., Hughes S.G., Gaudet D., Hegele R.A., O’Dea L.S.L. (2021). Efficacy and safety of volanesorsen in patients with multifactorial chylomicronaemia (COMPASS): A multicentre, double-blind, randomised, placebo-controlled, phase 3 trial. Lancet Diabetes Endocrinol..

[52] U.S. Food and Drug Administration Tryngolza. https://www.accessdata.fda.gov/drugsatfda_docs/label/2024/218614s000lbl.pdf.

[53] Packard C.J., Pirillo A., Tsimikas S., Ference B.A., Catapano A.L. (2024). Exploring apolipoprotein C-III: Pathophysiological and pharmacological relevance. Cardiovasc. Res..

[54] Stroes E.S.G., Alexander V.J., Karwatowska-Prokopczuk E., Hegele R.A., Arca M., Ballantyne C.M., Soran H., Prohaska T.A., Xia S., Ginsberg H.N. (2024). Olezarsen, Acute Pancreatitis, and Familial Chylomicronemia Syndrome. N. Engl. J. Med..

[55] Bergmark B.A., Marston N.A., Prohaska T.A., Alexander V.J., Zimerman A., Moura F.A., Murphy S.A., Goodrich E.L., Zhang S., Gaudet D. (2024). Olezarsen for Hypertriglyceridemia in Patients at High Cardiovascular Risk. N. Engl. J. Med..

[56] Tardif J.-C., Karwatowska-Prokopczuk E., Amour E.S., Ballantyne C.M., Shapiro M.D., Moriarty P.M., Baum S.J., Hurh E., Bartlett V.J., Kingsbury J. (2022). Apolipoprotein C-III reduction in subjects with moderate hypertriglyceridaemia and at high cardiovascular risk. Eur. Heart J..

[57] Watts G.F., Rosenson R.S., Hegele R.A., Goldberg I.J., Gallo A., Mertens A., Baass A., Zhou R., Muhsin M., Hellawell J. (2025). Plozasiran for Managing Persistent Chylomicronemia and Pancreatitis Risk. N. Engl. J. Med..

[58] Gaudet D., Pall D., Watts G.F., Nicholls S.J., Rosenson R.S., Modesto K., San Martin J., Hellawell J., Ballantyne C.M. (2024). Plozasiran (ARO-APOC3) for Severe Hypertriglyceridemia: The SHASTA-2 Randomized Clinical Trial. JAMA Cardiol..

[59] Ballantyne C.M., Vasas S., Azizad M., Clifton P., Rosenson R.S., Chang T., Melquist S., Zhou R., Mushin M., Leeper N.J. (2024). Plozasiran, an RNA Interference Agent Targeting APOC3, for Mixed Hyperlipidemia. N. Engl. J. Med..

[60] Gaudet D., Greber-Platzer S., Reeskamp L.F., Iannuzzo G., Rosenson R.S., Saheb S., Stefanutti C., Stroes E., Wiegman A., Turner T. (2024). Evinacumab in homozygous familial hypercholesterolaemia: Long-term safety and efficacy. Eur. Heart J..

[61] Rosenson R.S., Gaudet D., Ballantyne C.M., Baum S.J., Bergeron J., Kershaw E.E., Moriarty P.M., Rubba P., Whitcomb D.C., Banerjee P. (2023). Evinacumab in severe hypertriglyceridemia with or without lipoprotein lipase pathway mutations: A phase 2 randomized trial. Nat. Med..

[62] Bergmark B.A., Marston N.A., Bramson C.R., Curto M., Ramos V., Jevne A., Kuder J.F., Park J.-G., Murphy S.A., Verma S. (2022). Effect of Vupanorsen on Non-High-Density Lipoprotein Cholesterol Levels in Statin-Treated Patients With Elevated Cholesterol: TRANSLATE-TIMI 70. Circulation.

[63] Zimerman A., Wiviott S.D., Park J.-G., Murphy S.A., Ran X., Bramson C.R., Curto M., Ramos V., Jevne A., Kuder J.F. (2024). Hepatic fat changes with antisense oligonucleotide therapy targeting ANGPTL3. J. Clin. Lipidol..

[64] Zimerman A., Wiviott S.D., Park J.-G., Murphy S.A., Ran X., Bramson C.R., Curto M., Ramos V., Jevne A., Kuder J.F. (2024). Reductions in remnant cholesterol and VLDL cholesterol through inhibition of ANGPTL3 protein synthesis: An analysis from the TRANSLATE-TIMI 70 trial. Eur. J. Prev. Cardiol..

[65] Rosenson R.S., Gaudet D., Hegele R.A., Ballantyne C.M., Nicholls S.J., Lucas K.J., San Martin J., Zhou R., Muhsin M., Chang T. (2024). Zodasiran, an RNAi Therapeutic Targeting ANGPTL3, for Mixed Hyperlipidemia. N. Engl. J. Med..

[66] Bailey N.N., Peterson S.J., Parikh M.A., Jackson K.A., Frishman W.H. (2023). Pegozafermin Is a Potential Master Therapeutic Regulator in Metabolic Disorders: A Review. Cardiol. Rev..

[67] Geng L., Lam K.S.L., Xu A. (2020). The therapeutic potential of FGF21 in metabolic diseases: From bench to clinic. Nat. Rev. Endocrinol..

[68] Kliewer S.A., Mangelsdorf D.J. (2019). A Dozen Years of Discovery: Insights into the Physiology and Pharmacology of FGF21. Cell. Metab..

[69] Chui Z.S.W., Shen Q., Xu A. (2024). Current status and future perspectives of FGF21 analogues in clinical trials. Trends Endocrinol. Metab..

[70] Bhatt D.L., Bays H.E., Miller M., Cain J.E., Wasilewska K., Andrawis N.S., Parli T., Feng S., Sterling L., Tseng L. (2023). The FGF21 analog pegozafermin in severe hypertriglyceridemia: A randomized phase 2 trial. Nat. Med..

[71] Hartsfield C., Bhatt D., Bays H., Maki K., Feng S., Agollah G., Mansbach H., Kastelein J., Parli T. (2024). Study Design of a Phase 3 Randomized Controlled Trial Evaluating the Efficacy and Safety of Pegozafermin in Patients with Severe Hypertriglyceridemia. J. Clin. Lipidol..

[72] Albekery M.A., Alhomoud I.S., Alabdulathim L.S., Almajed M.A., Alobaid A.A., Alomair M.K., Al Shehab S.A., Alhasan K.A., Al Hamid A. (2025). Underutilization of albuminuria screening in adults with diabetes mellitus or hypertension: A systematic review and meta-analysis. BMC Nephrol..

[73] Altuwalah A., Alfehaid L., Alhmoud H., Alrasheedy A., Alhomoud I. (2025). ApoB Testing in Dyslipidemia Management: Knowledge and Practices of Healthcare Providers in Saudi Arabia. JMDH.

[74] Alhomoud I.S., Talasaz A.H., Chandrasekaran P., Brown R., Mehta A., Dixon D.L. (2024). Incretin hormone agonists: Current and emerging pharmacotherapy for obesity management. Pharmacotherapy.

[75] Alhomoud I.S., Wheeler S.E., Dixon D.L. (2025). Repurposing Incretin Therapies: A Narrative Review of Emerging Indications Across Cardiometabolic, Liver, Kidney, Neurological, Psychiatric, and Other Systems. Can. J. Physiol. Pharmacol..

[76] Oral E.A., Garg A., Tami J., Huang E.A., O’Dea L.S.L., Schmidt H., Tiulpakov A., Mertens A., Alexander V.J., Watts L. (2022). Assessment of efficacy and safety of volanesorsen for treatment of metabolic complications in patients with familial partial lipodystrophy: Results of the BROADEN study. J. Clin. Lipidol..

